# Acute Myocardial Infarction in the Era of COVID-19: A Challenge in a Challenge

**DOI:** 10.3390/jcm12185779

**Published:** 2023-09-05

**Authors:** Annamaria Mazzone, Sergio Berti, Cristina Vassalle

**Affiliations:** 1 Diagnostic and Interventional Department of Cardiology, Ospedale del Cuore, G. Monasterio Tuscany Foundation (FTGM), 56124 Massa, Italy; annamaria.mazzone@ftgm.it (A.M.);; 2 Fondazione CNR-Regione Toscana G Monasterio, 56124 Pisa, Italy

From the very beginning, the coronavirus pandemic (COVID-19) has tested the healthcare systems, having unpredictable and extreme adverse impacts on acute care clinical settings. In particular, the pandemic led to new challenges in the diagnosis and treatment of acute ST elevation myocardial infarction (STEMI). During the COVID-19 pandemic, a significant reduction in hospitalizations due to acute myocardial infarction was observed worldwide [1,2]. Patients frequently arrived at hospital too late, when the optimal time window for emergency primary percutaneous coronary intervention had already passed: at this stage, patients often present with cardiovascular complications and adverse outcomes [3,4]. The reasons for this trend are multifactorial and include lockdown measures; difficulties in consulting physicians, contacting the hospital, or calling an emergency service; reluctance to go to the hospital and fear of infection [4]. Thus, it is necessary to identify significant predictors of mortality and critical complications, considering both specific cardiovascular (CV) risk profiles and the features of SARS-CoV-2 infection, to prevent major adverse outcomes due to delayed hospitalization and in-hospital adverse events.

The five clinical studies collated in this Special Issue address a range of essential research topics related to STEMI in the COVID-19 time, including the characteristic profile of patients with acute coronary syndromes (ACS) during the pandemic, mortality or other major adverse outcomes, and various complications related to delayed hospitalization.

In their study, Popa-Fotea et al. [5] compared the profiles and outcomes of STEMI patients hospitalized during the pandemic compared with a control group of STEMI patients who were admitted to the emergency department in the pre-pandemic period. STEMI hospital admissions fell during the COVID-19 period during the first and second years of the pandemic (−30.26% and −25.4%, respectively), alongside a significant increase in all-cause in-hospital mortality (+11.5% in the pandemic period compared to +8.1% in the previous pre-COVID year). In conclusion, a significant relationship between SARS-CoV-2 infection and overall-cause in-hospital mortality was found, but no significant association was observed between COVID-19 and revascularization. Moreover, the profile of STEMI patients did not change over time, as they had similar demographic characteristics and comorbidities in both the pre-pandemic and pandemic eras.

To identify significant predictors of mortality and critical complications, Milovančev et al. aimed to evaluate the profiles and adverse events of COVID-19 patients with ACS who were hospitalized in two Serbian university centers [6]. Their results reveal more underlying comorbidities (hypertension, diabetes, chronic kidney disease, and previous percutaneous coronary intervention), adverse presenting features (e.g., acute heart failure), complications (including arrhythmias, cardiogenic shock, cardiopulmonary cerebral resuscitation, and left ventricle thrombus) and intrahospital 30-day mortality in COVID-infected patients compared with patients not infected. Thus, efforts should be focused on subjects with specific CV risk profiles to prevent SARS-CoV-2 infection where possible, or at least early recognized and appropriately evaluate and treat these patients as soon as possible.

The study by Yang et al. [7] aimed to assess the effects of the COVID-19 pandemic on Chinese patients with STEMI (in-hospital mortality, odds of primary percutaneous coronary intervention, and timely reperfusion), by using a nationwide database (the Chinese Cardiovascular Association Database–Chest Pain Centers). The authors found a persistent residual pandemic impact on STEMI management after the first wave of the COVID-19 pandemic in the Hubei area (the worst-affected region of China during this first wave) and a less evident impact in other Chinese provinces.

Altobelli et al. performed a meta-analysis to assess the admission trends of patients with STEMI/non-ST-segment elevation MI (NSTEMI) during the first pandemic wave, analyzing the risk profile and the occurrence of early adverse events [8]. Compared with the pre-COVID period, an significant decrease in hospitalizations with effects on early survival was observed; the time from symptom onset to first medical contact was particularly extended, whereas the increase in door-to-balloon time was much less significant. Regarding NSTEMI, the authors also observed a significant reduction in hospitalizations and a higher number of in-hospital deaths during the pandemic period. The authors hypothesized fear of infection and an underestimation of symptoms as reasons for the delay in patients presenting to hospital during the COVID-19 pandemic compared with the pre-COVID period, with a emphasis on the time from symptom onset to first medical contact. Instead, despite the logistic and technical difficulties for patients and healthcare professionals subjected to additive procedures and device use, the time from hospital admission to cath lab only marginally increased, leading to effective healthcare. Consequently, patients must be provided with effective and simple information in order to raise awareness of ischemic symptoms that should not be ignored and the serious consequences that may arise if they are not taken into account.

Marotta et al. studied levels of distress in healthcare professionals due to the overall critical situation faced during COVID-19 [9]. Their study assessed baseline distress in healthcare professionals before and after the COVID-19 pandemic, and the role of mindfulness-based stress reduction training on well-being, stress, and burnout. Although the lack of change in the baseline distress of healthcare professionals evaluated before and during the COVID time suggested a stronger influence of the type of work rather than by the pandemic itself, MBSR training lowered the distress levels of the workers, suggesting that this practice could be an essential component of general worker training as well as representing an effective approach to reduce distress in emergency periods.

The editors wish to thank authors who studied current and newly emerging topics in these challenging times, evaluating both personal- and system-related factors, which represent obstacles for both patients and healthcare workers. As a warning for potential future public health emergencies, it is essential to clearly communicate to patients the importance of timely interventions for acute myocardial infarction. This will avoid delays in or withdrawal from necessary medical care, as targeted measures can be adopted to benefit more vulnerable patients and those at high risk of CV diseases, as well as distressed healthcare professionals, optimizing the quality of healthcare.
